# Accuracy of triangular meshes of stone models created from DICOM cone beam CT data

**DOI:** 10.1186/s40729-019-0171-9

**Published:** 2019-05-08

**Authors:** Dimitrios Apostolakis, Georgios Michelinakis, Georgios Kourakis, Emmanuel Pavlakis

**Affiliations:** 1 Dental Radiology in Crete, Plateia 1866 39, 73100 Chania, Crete Greece; 2 Crete Implants, Heraklion, Greece; 3 Prosthetiki Dental Lab, Heraklion, Greece

**Keywords:** Mesh, Stereolithography, Cone beam computed tomography, Dental model

## Abstract

**Background:**

The aim of this study was to assess the theory that CBCT scanners can be used for a subsequent triangular mesh generation which accurately represents the actual stone model.

Ten, recently acquired stone models, were used in the present study. The stone models were initially scanned with the Dental Wings 7Series dental scanner. Each stone model was then scanned using a 150-μm voxel resolution in a Planmeca Mid CBCT device with 2 sets of exposure parameters and in a Newtom VG device. The DICOM files were initially imported in Blue Sky Plan implant surgery software, segmented and then imported for computational manipulation in CloudCompare, a dedicated mesh handling software.

**Results:**

For all CBCTs and for all exposure parameters, the mean (SD) difference was 0.052 (0.011) mm ranging from 0.032 to 0.070 mm with a 95% CI for the population mean of 0.052 ± 0.004 mm. Specifically, the mean (SD) difference for each device/exposure parameter tested was (1) Newtom VG = 0.040 (0.006) mm, (2) Planmeca Mid 90 = 0.057 (0.0066) mm, and (3) Planmeca Mid 80 = 0.059 (0.0063) mm.

**Conclusions:**

There are differences amongst the CBCT models, whilst different exposure parameters of the same model do not seem to offer a significant advantage. The interaction between the threshold value and the imaging modality as far as the errors are concerned necessitates the careful selection of the right threshold value for the triangular mesh creation.

## Background

The need for digital dental models is rising along with the increased usage of computer-aided designed and computer-aided manufactured (CAD/CAM) implant surgical guides, orthodontic clear aligners, the practice of 3D orthognathic surgery including the applications of the so-called virtual patient, and finally as a space-effective mean of model storage [1–7].

Scanning the stone model with either a 3D laser or a white light desktop scanner remains the gold standard for the digitisation procedure since this technique is widely accepted as being the best available method [8, 9]. However, these desktop scanners are usually situated in dental laboratories and not in dental offices.

Cone beam CT devices for dental usage are becoming increasingly common in dental practices, offering advanced diagnostic capabilities to the general and to the specialised practitioner with a reduced radiation dose for the patient, compared to medical multi-slice CT scanners [10–12].

In view of the increased costs related to the acquisition of a highly accurate desktop scanning device and the fact that CBCT devices are becoming increasingly common either in dental practices or in specialised dental radiological facilities, it is our theory that these CBCT scanners can be used for a subsequent triangular mesh generation that accurately represents the actual stone model. The expected effect size (differences between the two modalities, CBCT and desktop scanner) should not prohibit the effective usage of this mesh model in certain clinical situations.

In this study, three (3) null hypotheses were tested concerning the triangular meshes produced by the transformation of CBCT data in the form of the digital imaging and communications in medicine standard (DICOM) and the triangular meshes produced by a desktop scanner: (a) There is no significant difference amongst the tested CBCT scanners in relation to accuracy. (b) There is no significant difference between different exposure factors on the same model of the CBCT scanner in relation to accuracy. (c) There is no interaction of the CBCT imaging modality with the threshold value used for the conversion of DICOM data to STL meshes in relation to accuracy.

## Methods and methods

Ten (10), recently acquired for orthodontic reasons from fully dentate adult patients’ stone models, were used in the present study. The stone models (five maxillae, five mandibles) were casted from type IV dental stone (Hera Moldastone CN, Kulzer) using common laboratory procedure and were scanned with the different modalities (laser scanner and CBCTs).

**Fig. 1:**
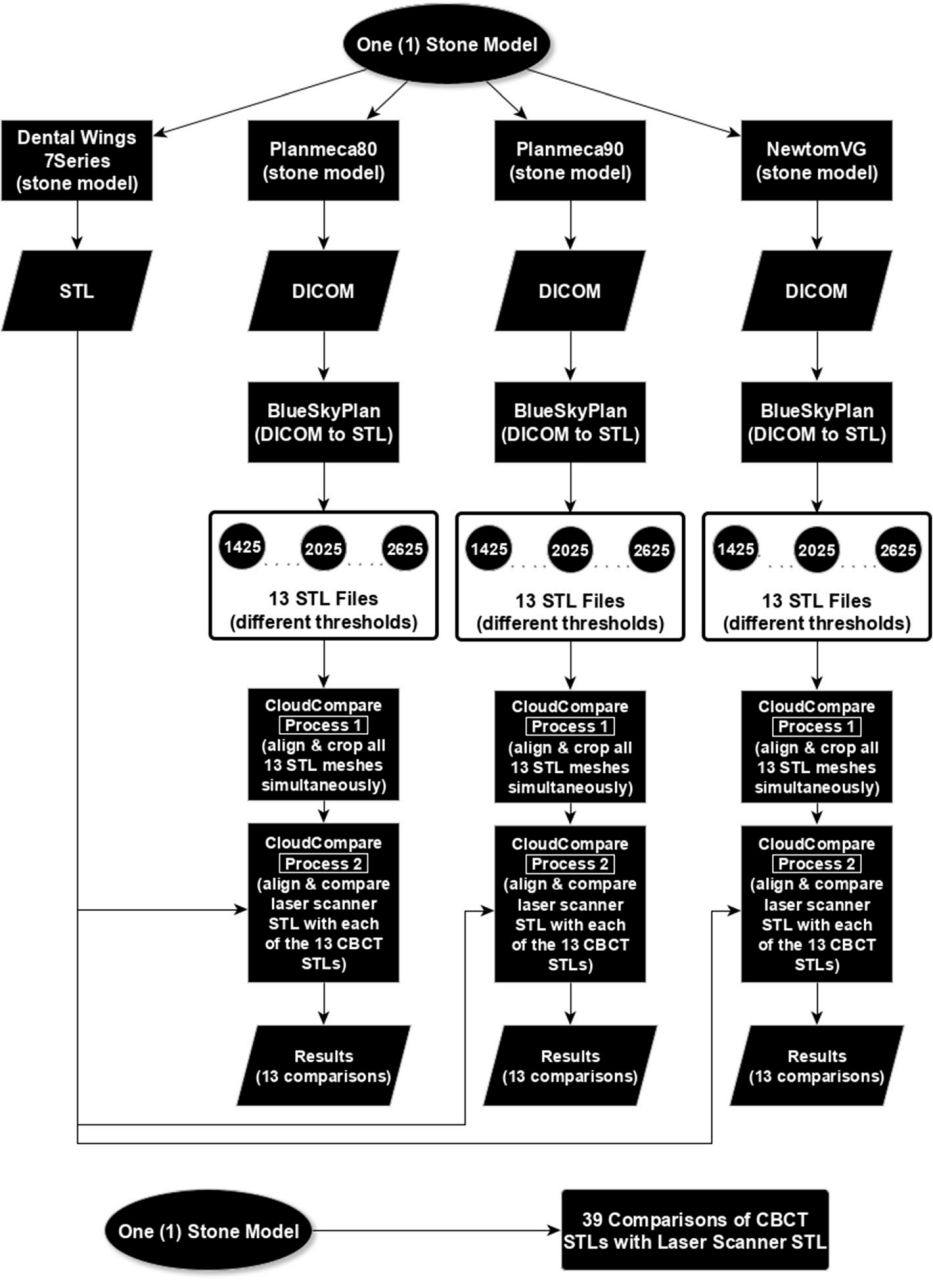
A diagram of our method

The Dental Wings 7series laser desktop scanner (Dental Wings Inc., Canada) was used as a reference scanner against which the meshes coming from CBCT devices were compared. This device is a commercial scanner equipped with a blue illumination laser beam used in dental labs and commonly employed for the digitisation of stone models in order to design and manufacture CAD/CAM dental prostheses. Its accuracy, as reported by the manufacturer, is 15 μm. The resultant triangular meshes of the stone models (stereolithography-STL files) were used as the gold standard (Fig. 1).

In order to estimate the repeatability of the reference scanner, the first stone model (case 1, maxilla) was scanned with the laser scanner ten times. The ten meshes were simultaneously cropped and were finely registered with each other, following the same procedure as later described for the main study. This resulted in 90 pairs of meshes whilst each of the meshes acquired was sequentially used as a reference. The average standard deviation of the differences of the meshes was used as a measure of repeatability for the desktop scanner.

Each stone model was then scanned using a 150-μm voxel resolution:

1. With a Planmeca Mid CBCT device (80 KVp, 12.5 mA, 15 s): ‘Planmeca80’
2. With the same Planmeca Mid CBCT (90 KVp, 14 mA, 15 s): ‘Planmeca90’
3. With a Newtom VG device (110 KVp, auto exposure mode): ‘Newtom VG’

It should be noted that the scanning sequence started within 2 days after the desktop laser scanning, initially by using the Planmeca Mid (80 KVp, 12.5 mAs) device which was immediately followed by x-ray scanning with the same Planmeca Mid but with different exposure parameters (90 KVp, 14 mAs). The Newtom VG x-ray scanning commenced approximately 15 days later since this device is situated in another facility.

The data were exported in DICOM format by the proprietary software of each scanner resulting in three DICOM data folders for each stone model.

The DICOM files were then imported into the Blue Sky Plan implant surgery software (Blue Sky Plan, Blue Sky Bio, USA) and were segmented using the segmentation tool for models, incorporated in the software. The window level (L) was initially set at the value of 1425 and increased up to the value of 2625 in 100 unit steps in a Hounsfield (HU) scale with a scale range from − 1000 to 7000 units. The window width (W) is automatically set by the software to 2500 units. This manipulation resulted in 13 different segmentations for each stone model. Triangular meshes were calculated and exported as STL files for each of these segmentation values. As a result, 39 STL stone model representations for each stone model and for all radiation scanning modalities were produced.

For each stone model, the 40 (reference standard and CBCTs) meshes were imported for computational manipulation in a dedicated mesh and point cloud handling software (CloudCompare, http://www.cloudcompare.org/). The triangular mesh which derived from laser scanning was used as a reference, and no other manipulation was permitted. The 39 CBCT-originated meshes were then initially roughly registered together using a minimum (3–5) number of points and then were again finely registered with each other using the iterative closest point (ICP) algorithm, calculated on a sample of 50,000 pairs of points. This resulted in the 39 meshes for each stone model overlapping one another. The meshes were then simultaneously cropped, thus leaving only the teeth and 3–5 mm of the gingiva. The end result was 39 triangular meshes representing the same stone model, with clinically relevant remaining anatomy, almost identical for each mesh. Finally, each of these meshes was again separately, roughly, and finely registered to the reference laser model (gold standard). This resulted in 39 registered to the gold standard different meshes for each stone model.

For each registered to the gold standard CBCT mesh, the absolute distance of each and every face of the mesh to a point on the surface of the reference (gold) standard was computed indicating the difference that exists between this mesh and the gold standard. The median value of the differences and the interquartile range (IQR) for each pair was noted. The product of the median value and the IQR was computed and was used as an index for the best matching mesh pair between the 13 pairs that constituted the group of segmentations for each stone model per scanning modality. We named this product the Dissimilarity Index (DI):

$$ \mathrm{Dissimilarity}\ \mathrm{Index}=\mathrm{median}\times \mathrm{interquartile}\ \mathrm{range}\times 1000 $$

The Dissimilarity Index (DI) ranges from zero to infinity with the values closer to zero representing smaller overall differences between the mesh coming from the CBCT and the gold standard.

In order to estimate the accuracy and the precision of our registration software (CloudCompare), five laser-scanned meshes were used. Each mesh was imported in the software and cloned; the clone was moved in a random way and then roughly and finely registered with the original. The mean difference between the meshes was used as a measure of the software accuracy whilst the standard deviation of the differences was used as a measure of the registration precision of the software.

Descriptive statistics were calculated, and inferences were drawn using (a) repeated measures one-way ANOVA with fixed factor ‘X-ray Modality’ (i.e. Planmeca80, Planmeca90, Newtom VG) and dependent variable ‘the lowest (= best) value of the Dissimilarity Index per stone case and per x-ray modality (30 values)’ and (b) two-way ANOVA with fixed factors ‘X-ray Modality’ and ‘Threshold value’ and dependent variable ‘the Dissimilarity Index’, with Greenhouse-Geisser correction if appropriate (390 values). Results were considered significant for a Dunn- Sidak corrected *p* < 0.05. SPSS version 20 was used for the analysis. All values were rounded to 2 significant digits.

## Results

The results for the repeatability study of the reference scanner and those for the accuracy and repeatability of the mesh handling software are presented in Table 1.

**Table 1:** Repeatability results for the reference scanner and the CloudCompare software

	SD	Mean
Laser scanner		
*N* = 90	0.0041	0.016
Mesh handling software		
*N* = 5	0.000046	0.000001

For the pair of meshes with the lowest (best) Dissimilarity Index for every stone model case and for every x-ray modality, the threshold value, the value below which the 95% of the differences between all the points are included, the median value of the error, and the IQR and the DI value are presented in Table 2. For these cases, for all CBCTs and for all exposure parameters (30 measurements), the mean (SD) difference was 0.052 (0.011) mm ranging from 0.032 to 0.070 mm with a 95% CI for the population mean of 0.052 ± 0.004 mm.

**Table 2:** Various statistics for the CBCT meshes with the lower (best) Dissimilarity Index per case

No. of stone models = 10	Threshold	< 0.95(mm)	Median (mm)	IQR (mm)	DI (mm^2^)
Planmeca80	2425	0.18	0.051	0.062	3.2
	2225	0.19	0.057	0.076	4.3
	2325	0.19	0.055	0.073	4.0
	2125	0.19	0.065	0.078	5.1
	2025	0.17	0.053	0.063	3.3
	2325	0.15	0.053	0.068	3.6
	1925	0.20	0.069	0.082	5.7
	2225	0.16	0.056	0.070	3.9
	2025	0.18	0.067	0.080	5.3
	1925	0.21	0.060	0.080	4.8
Planmeca90	2425	0.20	0.051	0.061	3.1
	2325	0.18	0.050	0.080	4.0
	2225	0.16	0.057	0.073	4.1
	2225	0.19	0.052	0.070	3.6
	2125	0.20	0.063	0.084	5.3
	2325	0.17	0.053	0.063	3.3
	2025	0.16	0.057	0.076	4.3
	2025	0.19	0.063	0.078	4.9
	1925	0.18	0.070	0.080	5.6
	1825	0.21	0.058	0.078	4.5
NewtomVG	2225	0.13	0.045	0.063	2.8
	2025	0.12	0.035	0.049	1.7
	1925	0.13	0.043	0.053	2.3
	2025	0.13	0.039	0.049	1.9
	1825	0.14	0.044	0.058	2.5
	2125	0.10	0.032	0.039	1.2
	2025	0.11	0.032	0.047	1.5
	1925	0.13	0.035	0.052	1.8
	1825	0.16	0.049	0.065	3.2
	1825	0.16	0.045	0.051	2.3

For the Planmeca Mid (80 KVp, 12.5 mAs), the mean difference (SD) was 0.059 (0.0063) mm with a 95% confidence interval of 0.059 ± 0.0045 mm.

For the Planmeca Mid (90 KVp, 14 mAs), the mean difference (SD) was 0.057 (0.0066) mm with a 95% confidence interval of 0.057 ± 0.0047 mm.

For the Newtom VG (110 KVp, auto exposure), the mean difference (SD) was 0.040 (0.006) mm with a 95% confidence interval of 0.040 ± 0.0043 mm.

One-way repeated measures ANOVA revealed that differences exist between the 3 imaging modalities (*F*(2, 18) = 30.17, *p* < 0.0005). Pairwise comparisons elicited that the Newtom VG device had a significantly smaller error from the Planmeca80 (mean difference = 0.017 mm, *p* < 0.0005, 95% CI 0.009–0.028 mm) and from the Planmeca90 (mean difference = 0.0019 mm, *p* < 0.0005, 95% CI 0.011–0.023 mm), whilst the errors between the 2 Planmeca sets of exposure parameters were not statistically significant (*p* = 1.00).

**Fig. 2:**
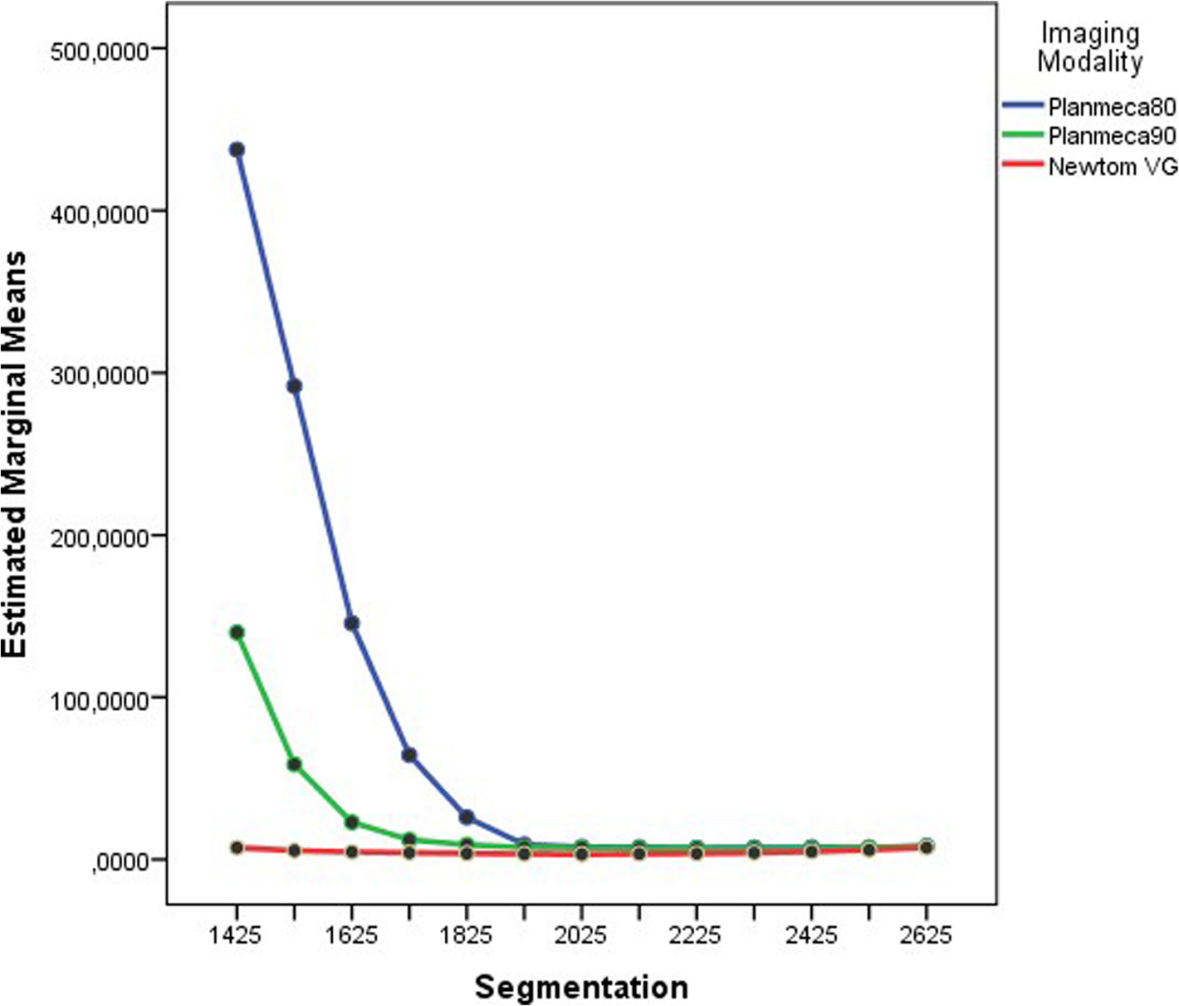
A plot of the threshold values used for the segmentation of the stone models against the marginal means of the Dissimilarity Index for the different imaging modalities. An interaction between the imaging modalities and the segmentation value for the production of the error can be seen

Two-way repeated measures ANOVA with fixed factors ‘Xray Modality’ (3 levels) and ‘Threshold value’ (13 levels) was run. There was a statistically significant interaction between the factors (*F*(1.3, 11.5) = 18, *p* = 0.01), revealing that the effect of the imaging modality (Planmeca80, Planmeca90, Newtom VG) on the differences between the meshes (CBCT and gold standard) depends on the threshold value (Fig. 2). Therefore, simple main effects were run. For the simple main effect of x-ray modality on error, the differences between the Plamneca80 and Planmeca90 were significant for all the threshold values except the values 2025–2525 HU with the difference between the meshes (CBCT and gold standard) always positive (error Planmeca80 > Planmeca90). For the differences between the Planmeca80 and Newtom VG, and Planmeca90 and Newtom VG, the error was statistically significant for all the threshold values except the values 2525–2625 HU, with the Newtom VG exhibiting smaller error in all the threshold values (error Planmeca80 > Newtom VG and error Planmeca90 > Newtom VG) (Table 3).

**Table 3:** Simple main effects of x-ray modality on error

Threshold value (HU)	X-ray modality	Mean difference (DI)	Standard error (DI)	Sig.
1425	Plan80–Plan90	297	78	0.012
	Plan80–NewVG	430	93	0.004
	Plan90–NewVG	132	22	0.001
1525	Plan80–Plan90	233	56	0.007
	Plan80–NewVG	186	63	0.004
	Plan90–NewVG	53	12	0.004
1625	Plan80–Plan90	123	19	0.0005
	Plan80–NewVG	141	21	0.0005
	Plan90–NewVG	18	3.6	0.002
1725	Plan80–Plan90	52	9.1	0.001
	Plan80–NewVG	60	10	0.0005
	Plan90–NewVG	8	0.82	0.0005
1835	Plan80–Plan90	17	3.9	0.006
	Plan80–NewVG	22	3.7	0.001
	Plan90–NewVG	5	0.7	0.0005
1925	Plan80–Plan90	1.8	0.5	0.013
	Plan80–NewVG	5.8	0.55	0.0005
	Plan90–NewVG	4.1	0.23	0.0005
2025	Plan80–Plan90	0.44	0.29	*0.49*
	Plan80–NewVG	4.5	0.3	0.0005
	Plan90–NewVG	4.02	0.36	0.0005
2125	Plan80–Plan90	0.62	0.25	*0.11*
	Plan80–NewVG	4.05	0.45	0.0005
	Plan90–NewVG	3.4	0.44	0.0005
2225	Plan80–Plan90	0.46	0.16	*0.05*
	Plan80–NewVG	3.6	0.5	0.0005
	Plan90–NewVG	3.1	0.4	0.0005
2325	Plan80–Plan90	0.78	0.21	0.016
	Plan80–NewVG	3.2	0.56	0.001
	Plan90–NewVG	2.4	0.42	0.001
2425	Plan80–Plan90	0.78	0.36	*0.17*
	Plan80–NewVG	2.6	0.54	0.003
	Plan90–NewVG	1.9	0.39	0.003
2525	Plan80–Plan90	0.068	0.26	*1*
	Plan80–NewVG	1.5	0.6	*0.1*
	Plan90–NewVG	1.5	0.7	*0.216*
2625	Plan80–Plan90	0.35	0.5	*1*
	Plan80–NewVG	0.93	0.85	*0.92*
	Plan90–NewVG	0.58	0.86	*1*

Non-significant differences are indicated in italics. *DI* Dissimilarity Index

Various descriptive statistics are presented in Table 4.

**Table 4:** Various descriptive statistics

	Total	Planmeca80	Planmeca90	Newtom VG
No. of meshes	30	10	10	10
Mean threshold value^1^	2092	2155	2145	1975
Min threshold value^1^	1825	1925	1825	1825
Max threshold value^1^	2425	2425	2425	2225
SD^1^	184	177	193	135
95% CI^1^	2023–2161	2029–2281	2007–2283	1878–2072
Mean difference (mm)^2^	0.052	0.059	0.057	0.040
SD^2^	0.01	0.0063	0.0066	0.006
Min diffrence^2^	0.032	0.051	0.05	0.032
Max difference^2^	0.07	0.069	0.07	0.049
95% CI^2^	0.0040	0.0045	0.0047	0.0043
Mean < 95%^3^	0.17	0.18	0.18	0.13
Min < 95%^3^	0.1	0.15	0.16	0.10
Max< 95%^3^	0.21	0.21	0.21	0.16
Mean IQR^4^	0.067	0.073	0.074	0.052
Mean DI^5^	3.6	4.3	4.3	2.1

^1^The segmentation with the lowest Dissimilarity Index

^2^Statistics referring to the distribution of the median differences

^3^Values of differences for the 95% of the total number of points between the pairs of meshes (reference standard/CBCT)

^4^*IQR* interquartile range

^5^*DI* Dissimilarity Index

## Discussion

There is an increasing need for indirect digitisation of dental arches for simulation and treatment purposes. One method of indirect digitisation is scanning the cast model with a CBCT device to create triangular meshes which can then be used for the fabrication of certain dental prostheses. It was the main purpose of this study to estimate how accurately a CBCT scanner can replicate the details of the gypsum model, thus providing support to our theory coming from personal experience that CBCT scanners are capable of the task.

For the estimation of the repeatability of the reference scanner, the standard deviation of the differences between identical meshes was employed, a method also used by other studies [13, 14]. Our mean standard deviation between the 90 pairs of meshes was 4.1 μm, and the repeatability could be considered excellent. The mean value of 16 μm for the differences between the meshes is the average deviation from the truth (0 mm); it can be considered a measure of accuracy, and it is similar to the value provided by the manufacturer of the laser scanner (15 μm).

Instead of a commercial alternative, CloudCompare (Version 2.9 Omnia, http://www.cloudcompare.org/), an independent open source project and free software under the GNU General Public License, was chosen as our mesh handling and comparison software. The accuracy and the repeatability of the software as expressed by the standard deviation and the mean values of differences between identical meshes can be considered adequate for the needs of the present study (Table 1).

Concerning Blue Sky Plan (Blue Sky Bio, USA), the software used to calculate and export the triangular meshes from DICOM data, it is a commercial software used for the design and fabrication of 3D surgical implant guides. The segmentation is accomplished with the use of a Hounsfield calibrated scale, and the software allowed the easy export of multiple meshes of the same stone model in different and discrete threshold values. The range of threshold values was decided based on our experience of using the value of 1750 HU for the production of meshes in our practices.

**Fig. 3:**
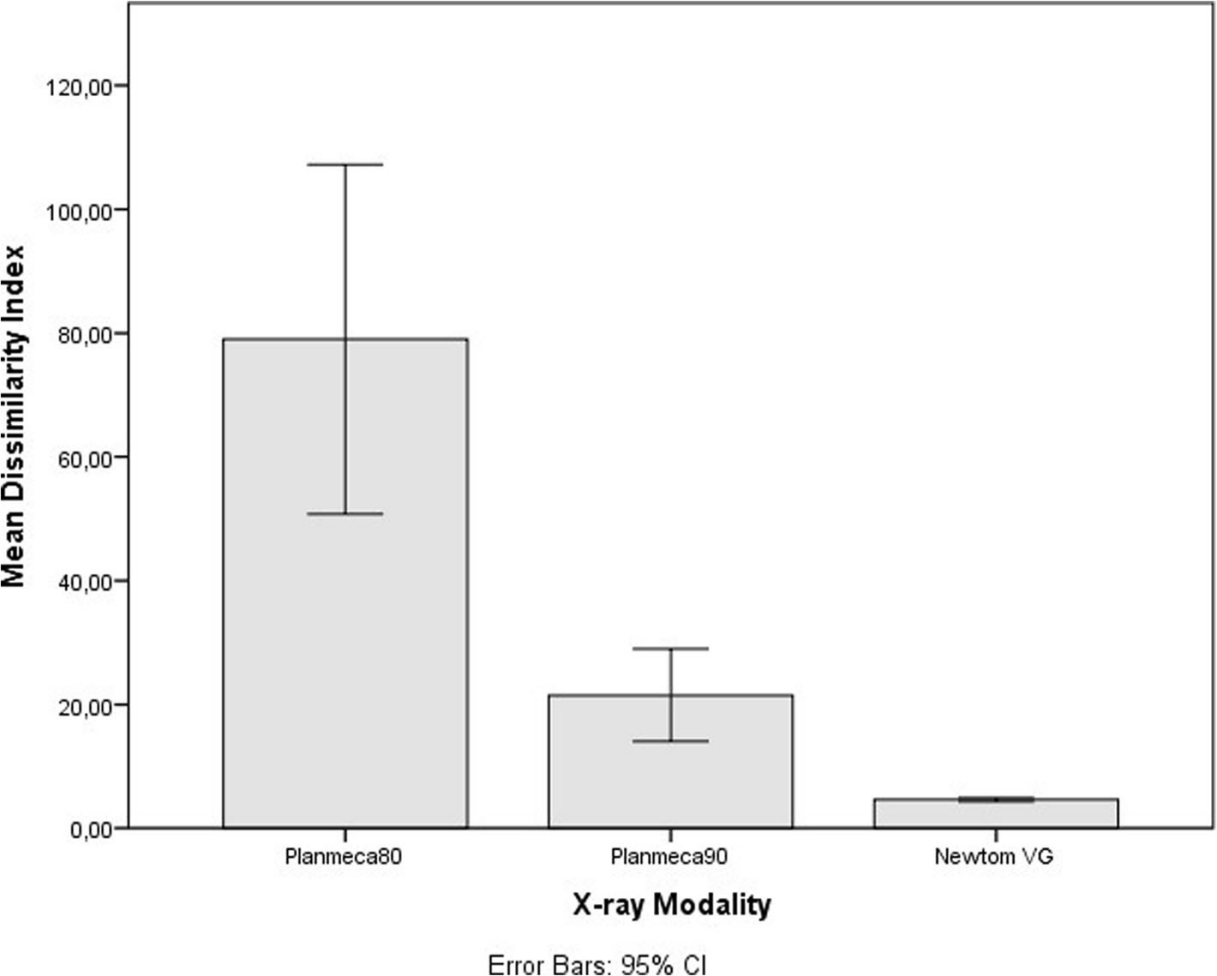
Error per x-ray modality

As the main statistic for our analysis, the Dissimilarity Index was used. This index can be considered as an unstandardized measure of effect and was devised when it became necessary to combine the measure of central tendency with the measure of the dispersion of the errors. Since the differences between the CBCT meshes and the gold standard were always skewed and away from normality, the median and the interquartile range were the appropriate statistics. It was invariably noted that the IQR values follow the tendency of the median (Fig. 3). However, when the median was approaching its lowest value, similar median values for different neighbour segmentations were computed. The product of the median and the IQR gives a greater resolution on the threshold value which best describes the pair of meshes with the lowest errors, taking into account not only the median value of the error but also the dispersion of the errors, too. The value of the DI can be easily traced back to its constituents (median and interquartile range), when necessary.

The average mean (SD) difference of the median errors for all the CBCT modalities was 0.052 mm (0.011) resulting in a 95% CI for the errors of 0.048 to 0.056 mm. To our knowledge, only one study evaluated the accuracy of stone model meshes originating from CBCT data [15]. The authors examined 8 different CBCT devices and found a mean difference of 0.064 ± 0.005 mm against 5 different extraoral digitisers used as a gold standard. However, in that study, the threshold value used for the DICOM to mesh conversion was at the discretion of the investigator. In our study, we found a significant interaction between the error and the threshold value for the different imaging modalities indicating that an appropriate threshold value must be computed for each device in order to minimise errors.

An average value of 0.052 mm for the median error must be evaluated taking into account the overall expected errors of other modalities that are used in order to digitise the tooth reality. The recommended in vitro benchmark of ± 20 μm for the replication of the tooth morphology establishes the upper limit of accuracy for the manufacturing of any successful prosthesis in the area of prosthodontics [16]. The in vitro accuracy of conventional impression and stone model pouring method has been found to be 20.4 ± 2.2 μm, and this error can be considered as the limit for the ability of the classical method to replicate reality [17]. The standard procedure for the indirect digitisation is the use of a lab desktop laser or white light scanner for the facilitation of CAD/CAM prostheses. The accuracy of these commercial lab scanners has been proven by a number of studies and ranges from 6 to 33 μm with the majority of the scanners in the under 20 μm category [18, 19]. A final and newer method for the direct replication of tooth anatomy is with the use of intraoral scanners that can entirely bypass the impression and stone model pouring technique. A number of studies show that the full-arch accuracy of these devices ranges in vitro from 11.5 to 332.9 μm. [17, 20–22].

The value below which the errors are situated when 95% of the points for each pair of meshes (CBCT and gold standard) is considered was also calculated. Even though values of central tendency and dispersion are useful, this 95% value gives an idea for the majority of the absolute errors that are expected in the totality of the stone model, without any averaging. This value being in any situation below 210 μm is conservative and, as it can be seen from inspection of the deviation maps, usually reflects the error from the soft tissue or is the result of calculations in a very small number of points. We removed the extreme 5% of the errors between points since it is expected that outliers can inflate our range of differences. It should be noted that in studies where differences between meshes are computed, 60–80% of the pairs of points are used [22]. In our study, the upper limit of the 95% of the values was in every case less than 210 μm and in the case of the Newtom VG device less than 150 μm.

Considering the different imaging modalities, the Newtom VG device was significantly more accurate than the Planmeca Mid for both sets of exposure parameters. Even though the stone models were scanned with the Newtom device 2 weeks after the stone pouring and suffered from handling due to transportation in another facility, the average error for the Newtom was 40 μm, ranging from a minimum value of 32 to a maximum value of 49 μm. The Newtom VG device operates in higher kilovoltage peak than the Planmeca, uses a rotating anode with a very small focal spot, and has a high total filtration value, which differences possibly partially explain its better performance. For the Planmeca Mid device, the difference between the two sets of exposure parameters was not significant, possibly implying that it is not the exposure parameters per se, but other software and hardware issues that increased the error compared to the Newtom VG. (Fig. 3).

A range of values was used in the Blue Sky Plan software in order to find the threshold that would produce the triangular mesh with the minimum error. The right threshold value is of importance especially for the Planmeca device since the error could be large for low threshold values. It was seen that with the low threshold value of 1425 HU, the error was maximum and the error reached a minimum at the area of 1825 to 2225 HU value after which it started increasing again, albeit very slowly. The mean best threshold value was almost the same for both Planmeca settings (2155 vs 2145 HU) as it was the range of the values for the smaller error (1925–2425 vs 1825–2425 HU) indicating that for the Planmeca device in general, any threshold value in the range of 1925–2425 HU can be used with relative safety. For the Newtom VG device, the errors in every threshold value were significantly less than the Planmeca and they reached their minimum at the values of 1825 to 2225 HU with a mean value of 1975 HU, indicating again that for Newtom VG device, any value in the range 1825–2225 HU can be used with relative safety. Combining the results of both CBCT models and of every exposure parameter, we find a mean (SD) value of 2092 (184) HU and a 95% CI for the mean of 2023–2161 HU. This range includes part of the best values of every device and every exposure setting and can be considered safe for the thresholding of stone models in all the CBCT devices of our study.

The use of CBCT for the scanning of stone models introduces artefacts to the final image. The divergent nature of the x-ray beam means that only the object lying in the midplane will be accurately reproduced [23]. Depending on the cone beam angle of the midplane, inaccuracy is to be expected due to data inconsistency. In the present study, the stone models were placed in the centre of the field of view and with the arch of the teeth parallel to the midplane minimising the image degradation. Beam hardening artefacts resulting from the polychromatic nature of the x-ray beam showing as streaks and shadows in the reconstructed images should also be expected, especially when the kilovoltage peak value is low [24]. In our cases, no such image degradation was optically noted for any given combination of exposure parameters, even when the 80 KVp tube voltage was used. Other possible limitations of the CBCT scanning method include the size of the focal spot and penumbra effects, the limited spatial resolution of the flat panel, the electronic and statistical x-ray noise, and the partial volume averaging effects [25]. In addition to the errors due to the physical process of x-ray exposure and the inherent limitations of the CBCT devices, there is always a possibility of having defects such as inaccuracy due to errors in the STL file, following the DICOM to STL conversion process [26].

With an average error of 0.052 mm, a number of applications seem feasible. In orthodontic applications, an error in the models of 200 μm seems acceptable [27], whilst in the cases of surgical guides, the knowledge of this error could be incorporated in the design, if necessary. For model storage, this value is more than adequate.

The main limitation of our study was the use of a commercial desktop dental scanner as our gold standard. The use of an industrial scanner could offer greater accuracy and repeatability. However, the repeatability of our scanner could be considered excellent, whilst the estimation of its accuracy was in the range defined by the manufacturer. In addition, the main purpose of our study was to estimate the performance of the x-ray scanners in comparison with a commonly used and universally available method.

In conclusion, the results of this study provide support to our theory that CBCT scanners can be used for the clinically relevant accurate digitization of stone models.

However, there are significant differences between the two CBCT models used; therefore, hypothesis ‘a’ was rejected. Different exposure parameters of the same CBCT model do not seem to offer a significant advantage, and, therefore hypothesis ‘b’ could not be rejected. Finally, the interaction between the threshold value and the exposure modality as far as the errors are concerned mandates the careful selection of the right threshold value for the triangular mesh creation. Tested hypothesis ‘c’ was therefore also rejected.

## Abbreviations

3D: Three-dimensional
CAD: Computer-aided design
CAM: Computer-aided manufacturing
CBCT: Cone beam computed tomography
CI: Confidence interval
DICOM: Digital Imaging and Communications in Medicine
STL: Surface tessellation language/stereolithography
